# Research Progress on Preparation, Transformation and Application of Protoplasts Derived from Medicinal Plants

**DOI:** 10.3390/plants15142227

**Published:** 2026-07-21

**Authors:** Zijin Fang, Yanheng Hu, Zijing Zhou, Huijie Ma, Lingxiao Zhang, Yuting Peng, Xiaori Zhan, Yiming Sun, Chenjia Shen

**Affiliations:** 1 College of Life and Environmental Sciences, Hangzhou Normal University, Hangzhou 311121, China; 2 College of Pharmaceutical Sciences, Zhejiang Chinese Medical University, Hangzhou 311402, China

**Keywords:** medicinal plant, protoplast, protoplast preparation, genetic transformation

## Abstract

As unicellular systems, protoplasts derived from medicinal plant cells exhibit high totipotency and hold significant value in applications such as gene function analysis, genetic improvement, and cell engineering optimization. This review focuses on how protoplast technology addresses longstanding bottlenecks in medicinal plant research by systematically collating recent advances in the study of medicinal plant protoplasts. It explores the multifaceted factors influencing protoplast preparation, including the intrinsic and extrinsic properties of medicinal plant materials, pretreatment methods prior to enzymatic hydrolysis, the composition of enzymatic solutions, enzymatic hydrolysis parameters, external environmental conditions, and protoplast purification techniques. Additionally, the review summarizes the significance and value of medicinal plant protoplasts in gene function verification, gene editing, genetic transformation, single-cell sequencing, and cell fusion regulation. By comprehensively synthesizing the optimization of the preparation of medicinal plant protoplasts and their application in transient expression, gene function research, and plant regeneration, this work aims to provide critical guidance for subsequent research in genetic modification, germplasm resource breeding, spatiotemporal programming of active substances, and regulatory network analysis. Ultimately, it serves as a valuable reference for advancing research in the plant sciences.

## 1. Dilemmas Faced in Medicinal Plant Research

Medicinal plants refer to plants containing one or more active ingredients, which usually have potential therapeutic value or serve as sources for drug isolation [1]. Among more than 1300 natural medicines used in Europe, 90% are derived from wild medicinal plant resources; of the top 150 prescription drugs used in the United States, approximately 118 originate from natural medicinal components [2]. Due to their rich content of various key bioactive substances, medicinal plants can be applied in healthcare maintenance, disease treatment, prevention, and other medical security aspects [3]. The accumulation of bioactive compounds in medicinal plants is not only regulated by internal physiological factors, such as variety, age, developmental stage, and organ differences, but also jointly affected by external environmental conditions, such as light, temperature, and geographical origin [3,4]. Due to common dilemmas, such as restrictions on the extraction of pharmacologically active ingredients, long growth cycles of plant materials, and difficulties in genetic transformation, studies on gene function verification, genetic improvement, and metabolic regulation of medicinal plants have been relatively backward [5,6,7,8]. The application of medicinal plant protoplasts helps to analyze the functions of genes related to the biosynthesis of active ingredients, which provide resources for genetic improvement. Furthermore, protoplast technology also realizes large-scale separation and extraction of active ingredients through regenerative culture, thus breaking through the limitations of conventional breeding [9].

Secondary metabolites in medicinal plants, which are characterized by low natural content, limited resources, and complex chemical structures, are the main pharmacodynamically active ingredients [7]. However, the synthesis and regulation mechanisms of active ingredients in medicinal plants remain unclear. The protoplast experimental technology provides a key path for overcoming environmental dependence in the breeding process, deepening functional analysis, and promoting the production of natural active ingredients [9]. Due to the unstable content of active ingredients in medicinal plants, researchers have established and optimized medicinal plant protoplast systems under artificially controlled environments. Furthermore, the protoplast system helps to create high-quality new germplasm, quickly analyze and regulate the biosynthesis pathway of active ingredients, and establish efficient single-cell regeneration and fast propagation systems.

Several medicinal plants have long life cycles, which brings many difficulties to their variety breeding and genetic improvement [5]. The rapid transient protoplast transformation system helps overcome the difficulty of the relatively long life cycles of some medicinal plants [10]. For decades, protoplasts have been used to expand genotypic diversity through interspecific and intraspecific hybridization. The medicinal plant protoplast systems play important roles in three terms: accelerating functional gene verification (*Ginkgo biloba* L., *Uncaria rhynchophylla*, *Eucommia ulmoides* Oliver, and *Taxus*), creating new germplasms (*Bletilla striata*, *Rhodiola sachalinensis*, and *Crocus sativus* L.), and providing materials for genetic improvement (*G*. *biloba*, *U*. *rhynchophylla*, *Macleaya cordata*, *Ilex rotunda* Thunb., *Lonicera japonica*, *Rh*. *sachalinensis*, and *B*. *striata*) [11,12,13,14,15,16,17,18,19,20,21,22]. Establishing an efficient genetic transformation system is crucial for analyzing the biosynthetic mechanisms of active ingredients in medicinal plants [23]. To overcome the common dilemma that most medicinal plants lack a mature genetic transformation system, a number of protoplast transformation systems have been established.

This paper systematically reviews the limitations of medicinal plants in active ingredient synthesis, cultivation cycle, and genetic transformation, summarizes the history, preparation methods, and application of medicinal plant protoplasts, aiming to break through the bottlenecks in medicinal plant research and provide new materials for genetic improvement and metabolic analysis.

## 2. Research History of Protoplast Preparation in Medicinal Plants

In 1880, Hanstein first proposed a protoplast, referring to the “living substance”, which is a plant cell whose cell wall can be completely or partially removed by mechanical or enzymatic methods [10,24]. Initially, protoplasts provided materials for basic research in physiology, biochemistry, and genetics [25]. Meanwhile, protoplasts are totipotent living cells that retain cellular characteristics and differentiation status, serving as excellent models for cell biology research [26].

Early studies on protoplasts were mostly focused on model plants or cash crops with clear genetic backgrounds [27]. In 1892, Klercker first used a mechanical method to remove the cell wall of algae to prepare protoplasts [28]. In 1960, British scientist Cocking successfully used an enzymatic method to efficiently and gently degrade the cell walls from young tomato root tips, obtaining a large number of highly active and regenerable protoplasts [29]. In 1970, Nagata and Takebe successfully prepared protoplasts from tobacco mesophyll tissue using an enzymatic method and created regenerated plants using protoplasts [30,31]. In 1972, Carlson et al. adopted protoplast fusion technology to successfully cultivate interspecific hybrids of the *Nicotiana* genus [32]. In 1978, Melchers et al. used PEG-induced fusion to incubate protoplasts of tomato and potato, successfully obtaining intergeneric hybrids [33]. The above three protoplast fusion studies overcame the incompatibility of distant hybridization and provided a technical paradigm for subsequent variety improvement and germplasm optimization.

In 1975, Gosch et al. isolated protoplasts derived from *Atropa belladonna* L. cell suspension cultures using an enzymatic method and established a regeneration system, marking the beginning of medicinal plant protoplast research [34]. In the same year, Schieder isolated protoplasts from *Datura innoxia* Mill. mesophyll cells [35]. Most medicinal plants contain high concentrations of secondary metabolites, such as phenols, alkaloids, and terpenoids, which easily interact with cell wall-degrading enzymes, affecting the efficiency of protoplast isolation [36]. Plant protoplasts can be isolated from various tissues, including leaves, calli, and suspension-cultured cells, while protoplast preparation mainly uses leaves as source materials [37]. Recently, great progress has been made in protoplast isolation from leaves of medicinal plants, such as *Areca catechu*, *Ricinus communis* L., and *Cannabis sativa* L. [38,39,40,41]. A small number of medicinal plants, such as *Angelica gigas* Nakai, *Chrysanthemum morifolium* ‘White ND’, and *Pinellia ternata* (Thunb.) Breit has completed whole-plant regeneration from protoplasts [25,42,43]. With protoplast fusion technology, intergeneric hybrids have been obtained from medicinal plants fused with model plants, such as combinations of *Nicotiana tabacum* L./*A*. *belladonna*, *Nicotiana undulata* L./*Lycium barbarum* L., *D*. *innoxia*/*A*. *belladonna*, and *Duboisia myoporoides/Duboisia hopwoodii* [44,45,46,47,48,49]. Gene editing has been successfully implemented in medicinal plant protoplasts, such as *Salvia miltiorrhiza* and *C*. *sativa* [24,50,51]. Medicinal plants undergo single-cell sequencing studies by preparing protoplasts, such as *Catharanthus roseus* and *Nepeta tenuifolia* [36,52]. However, the application of protoplasts derived from medicinal plants is still limited.

## 3. Preparation and Purification of Protoplasts from Medicinal Plants

### 3.1. Preparation of Protoplasts from Medicinal Plants

Protoplast preparation is the primary step for transformation, cell culture, and subsequent applications. The protoplast preparation process is susceptible to tissue physiology; therefore, a thin cell wall, easy enzymatic hydrolysis, and high homogeneity are regarded as the main points for protoplast preparation [37]. There are two main preparation methods: the mechanical method (also called the physical method) and the enzymatic method (also called the chemical method) [53].

**Figure 1:**
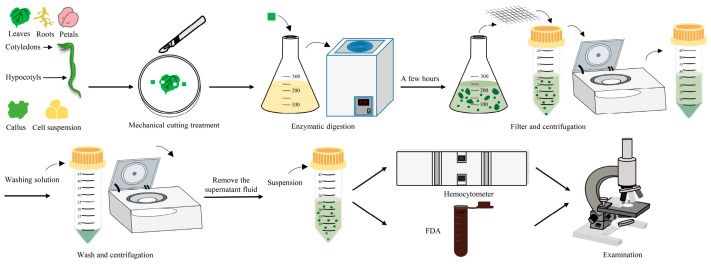
Schematic workflow of protoplast isolation from medicinal plants. The general procedures for medicinal plant protoplast preparation consist of multiple sequential steps, including plant material pretreatment, enzymatic digestion of cell walls, crude filtration, centrifugal precipitation, rinsing and resuspension, as well as the detection of protoplast yield and viability, followed by microscopic observation.

In early studies, the mechanical method was the primary approach for protoplast preparation [54]. Due to the limitations in cell type, operational difficulty, and osmotic pressure control, the mechanical method yields protoplasts with low efficiency and causes significant cell damage, greatly limiting its application. The enzymatic method typically involves incubating plant materials in a mixed isotonic enzyme solution capable of degrading cell walls, and then collecting viable protoplasts after the plant cell walls are disrupted [29,55]. Cellulase and pectinase are the commonly used enzymes for dissociating protoplasts from medicinal plants by targeting and digesting the main components of plant cell walls [56]. Compared with the mechanical method, the enzymatic method has several advantages, such as mild reaction conditions, high cell yield and activity, good integrity, and minimal damage to living cells. However, it is also restricted by secondary metabolites (e.g., phenols, alkaloids, terpenoids) and the specificity of enzymes. Although the enzymatic method is more suitable for plant protoplast isolation (Figure 1), the existing limiting factors urgently need to be addressed.

### 3.2. Purification of Protoplasts from Medicinal Plants

After enzymatic hydrolysis, the sample usually contains a large amount of cellular components, such as organelles, cell debris, and protoplast fragments, which affect the viability and physiological state of protoplasts [56]. After enzymatic hydrolysis, the crude protoplast extract is generally filtered through a 40–400 mesh sieve, and the filtrate is collected for further purification by centrifugation [57]. The common purification methods for medicinal plant protoplasts mainly include centrifugation, flotation, and interface [58].

Protoplasts are sedimented by low-speed centrifugation of the filtrate (based on their high specific gravity), followed by supernatant aspiration and resuspension in washing buffer for collection [12]. This widely used method yields high protoplast numbers, but centrifugation speed must be optimized to balance quality and purity. This technology has been used to purify *Acanthopanax senticosus* protoplasts by low-speed centrifugation using W5 washing solution, to provide a material basis for obtaining GUS-positive seeds through the PEG-mediated transient expression system and somatic embryogenic pathway in the future [59]. Based on the difference in specific gravity between protoplasts and impurities, the flotation method uses a hypertonic washing solution to make the protoplast layer float on the liquid surface during centrifugation [60]. The flotation method can obtain protoplasts with high purity but impairs protoplast integrity, resulting in a low yield. A previous study successfully isolated *C*. *sativa* protoplast separation using a combination of W5 washing solution and 21% sucrose solution, to provide a large amount of material for subsequent subcellular localization analysis [40]. The interface method uses two solutions with significantly different osmotic concentrations, thereby forming a pure protoplast band at the liquid interface [12]. A typical application is the purification of *C*. *morifolium* ‘White ND’ protoplasts using sucrose solutions of CPW salt solution and different concentrations, and obtained a green floating protoplast band at the interface between sucrose and washing solution, successfully purifying the protoplasts, to provide single-cell materials for subsequent shot generation [42].

Impurity components from enzymatic hydrolysis vary among medicinal plant species, leading to differences in suitable protoplast purification methods and washing solution compositions. The advantages of centrifugation are minimal damage, a high recovery rate, and wide applicability. It is suitable for large-scale transformation culture of protoplasts and cell regeneration; flotation can only be used in small quantities for pre-experimental conversion; the interface method has high purity and can be widely used in single-cell research. It is more suitable for the transformation of small systems and the preparation of high-quality regenerated strains. Purified protoplasts with uniform morphology, quantity, and quality can be directly used for subsequent experiments.

### 3.3. Quality Evaluation of Medicinal Plant Protoplasts

High yield and viability are critical for protoplasts and their transient expression [61]. The yield of protoplasts from medicinal plants is typically determined using the hemocytometer method [16]. The hemocytometer method has been used to determine protoplast yield in various medicinal plants, such as *G*. *biloba*, *U*. *rhynchophylla*, *E*. *ulmoides*, *A*. *gigas*, *C*. *roseus*, *R*. *communis*, *C*. *sativa*, *C*. *morifolium*, *Camellia oleifera*, *A*. *senticosus*, *Phellodendron amurense* rupr., *Elettaria cardamomum* Maton., *Zingiber officinale* Rosc., *Lotus corniculatus* L., *Gynostemma pentaphyllum* (Thunb) Makino, *Dendrobium candidum* Wall. ex Lindl., *Codonopsis pilosula* (Franch.) Nannf., *Pinellia cordata* N.E.Br, and *Picrorhiza kurrooa* [11,12,13,14,25,36,39,40,42,58,59,62,63,64,65,66,67,68,69,70,71,72,73,74,75,76,77,78,79].

Protoplast viability detection mainly adopts the FDA staining method, which can specifically label living cells. This method has minimal impact on protoplast activity and basic metabolism, making it the most widely used method for detecting protoplast activity [66]. To date, the FDA staining method has been applied for protoplast viability detection in various medicinal plants, including *G*. *biloba*, *U*. *rhynchophylla*, *E*. *ulmoides*, *S*. *miltiorrhiza*, *C*. *roseus*, *A*. *catechu*, *R*. *communis*, *C*. *morifolium*, *C*. *oleifera*, *A*. *senticosus*, *C*. *sativa*, *P*. *amurense*, *L*. *corniculatus*, *D*. *candidum*, *P*. *cordata*, *P*. *kurrooa*, *Z. officinale*, *Astragalus melilotoides* Pall., *Taxus canadensis*, and *Solanum dulcamara* L. [11,12,13,14,24,36,38,39,42,58,59,63,64,66,67,69,70,72,75,76,77,78,79,80,81,82,83].

## 4. Main Factors Affecting the Preparation of Protoplasts from Medicinal Plants

The preparation of medicinal plant protoplasts is a complex process that involves the synergistic action of multiple parameters. Different preparation and purification schemes can alter the physiological state, yield, and activity of protoplasts, ultimately affecting their genetic transformation, gene editing, plant regeneration, and single-cell analysis for subsequent research.

### 4.1. Intrinsic Variables

#### 4.1.1. Species of Medicinal Plants

Before protoplast isolation, plant materials must be strictly screened to avoid using those plant tissues infected by pathogens or in poor growth conditions [84]. The properties of plant materials for protoplast isolation include internal factors, such as inherent genetic characteristics, age, and tissue origin, as well as external factors, such as temperature, light, and humidity, all of which affect the yield and activity of the isolated protoplasts [10].

Plant cells are encased by rigid, semipermeable cell walls made up of complex polysaccharide matrices [85]. Marked interspecific and inter-tissue variations in cell wall constituents, including cellulose and pectin contents, necessitate the selection of different cell wall-degrading enzymes, as well as adjustment and optimization of enzyme proportions and digestion conditions to maximize protoplast isolation efficiency [54,86]. Different medicinal plants have distinct genetic backgrounds, and even different varieties of the same species possess varied genotypes, which exert significant impacts on the protoplast preparation processes. Comparative variety experiments fully confirmed this genotype-dependent characteristic. Under identical experimental conditions, significant differences were observed in petal protoplast yield and activity among three *C*. *morifolium* cultivars, including ‘Zi Fengche’ [69].

#### 4.1.2. Growth State of Plant Materials

The growth state of plant materials serves as a key intrinsic variable determining protoplast isolation efficiency and quality in medicinal plants. For the plants using leaves as materials, mature leaf tissues can yield protoplasts with higher production and vitality [87]. Nevertheless, such a tissue-dependent pattern exhibits marked species-specificity due to inherent tissue traits. In the woody medicinal plant *Taxus*, only young needles can generate protoplasts, whereas mature needles fail to do so, although the protoplast yield from young needles remains relatively low [15]. Thus, the inherent genetic characteristics of the plant are crucial for successful protoplast isolation.

#### 4.1.3. Tissues of Plant Materials

The tissue source significantly affects the protoplast yield and activity. The tissue sources of plant materials include vegetative organs (e.g., leaf, root, stem), reproductive organs (e.g., petal), seed embryo structures (e.g., hypocotyl, cotyledon), and materials induced by tissue culture (e.g., calli, cell suspension). Leaves are the most commonly used tissue for protoplast isolation due to their easy accessibility, abundant tissue cells, ease of separation and experimental manipulation, and high reproducibility in protoplast preparation [37,88].

There is a tissue preference in selecting raw materials for protoplast preparation across different medicinal plants [89]. Young, vigorously growing tissue materials are more likely to yield high-activity protoplasts [56]. Moreover, friable embryogenic calli have a larger enzymatic hydrolysis contact area than other tissues; thus, they can achieve more efficient protoplast release, making them more ideal materials [90].

### 4.2. Process Variables

#### 4.2.1. Pretreatment Methods Before Enzymatic Hydrolysis

Prior to protoplast preparation, pretreatments are more conducive to inducing in situ dedifferentiation and disrupting the cell wall structure, thereby improving enzymatic hydrolysis efficiency and increasing protoplast yield [88]. The main pretreatment methods for medicinal plants include vacuum, dark, plasmolysis, low-temperature, and mechanical cutting treatment [91]. Vacuum and mechanical cutting belong to physical intervention-based pretreatment, whose main function is to break down the external barriers of plant tissues. Vacuum can drive the enzyme solution deep into the tissue under negative pressure, ensuring sufficient contact between the enzyme and the cells [91]. A vacuum treatment of 0.85 MPa for 30 min has been identified as an efficient condition for enzymatic digestion of *E*. *ulmoides* cell walls [14]. Mechanical cutting damages the cuticle of plant cell epidermis through peeling the epidermis, scrubbing with abrasives, or cutting organs into cross-sections, allowing enzymes for protoplast isolation to access the plant cell walls [88]. It serves as a universal pretreatment method suitable for the majority of medicinal plants. By contrast, dark, plasmolysis, and low-temperature treatment are categorized as physiological regulation-based pretreatments, with the core functions of protecting protoplast activity and reducing experimental damage. Dark treatment involves exposing plant materials to a short photoperiod to protect them from light damage and minimize the risk of photo-injury during protoplast isolation [56]. It is suitable for *Dendrobium nobile* protoplast isolation [92]. Plasmolysis with mannitol, sucrose, or their mixtures can achieve pre-separation of protoplasts from cell walls [56,57]. Comparative experiments between pre-plasmolysis treatment and non-pre-plasmolysis treatment have confirmed that this treatment significantly preserves *L*. *corniculatus* leaf protoplast viability [70]. Low-temperature treatment at 4 °C can alleviate mechanical and osmotic stress damage and increase the quantity and vitality of the isolated protoplasts [56]. A combined pretreatment of low-temperature dark incubation and 0.75 M mannitol immersion has been confirmed to significantly improve the yield and vitality of *B*. *striata* leaf protoplasts [16]. However, not all medicinal plant protoplast preparation studies require pretreatment. Studies have shown that high-quality protoplasts can be obtained from certain materials through simplified or even no pretreatment. For instance, protoplasts with high viability and integrity can be successfully isolated from *S*. *miltiorrhiza* leaves without any pretreatment, and mechanical cutting can be completely omitted during protoplast preparation of *Z*. *officinale* embryogenic cell suspensions [24,80]. Pretreatment also has certain side effects. Therefore, selecting an appropriate pretreatment method will optimize the efficiency and quality of medicinal plant protoplast preparation.

#### 4.2.2. Composition of Enzymatic Hydrolysis Solution

The optimal composition of the enzymatic hydrolysis solution for protoplast isolation varies among different medicinal plant materials. The key to protoplast preparation is selecting the appropriate cell wall-degrading enzymes based on differences in cell wall components across species [93]. Enzymes used for cell wall degradation mainly include Cellulase, Hemicellulase, and Pectinase, with friable or meristematic tissues primarily requiring Hemicellulase and Pectinase [56,88]. Additionally, using a single enzyme may result in partial degradation of the plant cell wall, leading to reduced protoplast yield [94]. Cellulase is often used in combination with Macerozyme, Driselase, and Pectolyase for protoplast isolation [42]. There are significant differences in the adaptability of various medicinal plant species with cell wall-degrading enzymes. Among the four commonly used enzymes, Macerozyme-R10 is the most suitable for the preparation of *E*. *ulmoides* protoplasts, with the best enzymatic hydrolysis efficiency [14]. In *C*. *sativa* leaf materials, adding Pectolyase significantly increased the yield of protoplasts [64].

Optimizing the enzyme solution concentration is another critical step in protoplast preparation [94]. Enzyme concentrations higher than 2% *w*/*v* can shorten enzyme incubation time, but usually damage the integrity of plant cell membranes and reduce the protoplast activity [88]. The gradient concentration test showed that treatment with 1.5% Cellulase-R10 yielded the highest *C*. *morifolium* ‘White ND’ leaf protoplasts production [42]. Systematic screening of enzyme combinations confirms that the mixed enzyme solution consisting of 2% Cellulase-R10, 0.5% Cellulase-RS, 0.5% Macerozyme-R10, 0.5% Hemicellulase, and 1% Pectinase can achieve efficient protoplast isolation from *A*. *melilotoides* embryogenic calli [81]. In summary, targeted optimization of enzyme species, combinations, and concentrations based on material characteristics is key to efficient protoplast preparation from medicinal plants.

#### 4.2.3. Enzymatic Hydrolysis Time and Rotation Speed

Enzymatic hydrolysis time is a core parameter for balancing the yield and viability of protoplasts [91]. Insufficient digestion time leads to incomplete cell wall degradation and low protoplast yield, whereas excessive enzymatic digestion causes cellular damage and reduces protoplast viability [14]. The time of enzymatic digestion required for protoplast release ranges from 4 to 10 h, depending on the type of plant material [95]. Young leaf tissues are suitable for shorter enzymatic digestion times. Research has found that 4–5 h of enzymatic hydrolysis achieved the highest protoplast yield and vitality of *S*. *miltiorrhiza* leaf protoplasts [24].

Mechanical shaking can accelerate the enzymatic hydrolysis and isolation process of plant protoplasts, and is an important physical parameter that affects the efficiency of protoplast enzymatic hydrolysis [88,91]. During plant protoplast digestion, the stirring speed of the enzyme solution on an orbital shaker typically ranges from 0 to 90 rpm, with an average of approximately 40 rpm [91]. Research has shown that gentle shaking resulted in a higher protoplast yield of *G*. *biloba* leaf protoplasts compared to static incubation [12].

Selecting appropriate enzymatic hydrolysis time and rotation speed based on plant species or tissue characteristics can, to a certain extent, improve the efficiency and quality of medicinal plant protoplast preparation.

#### 4.2.4. Temperature, pH, and Osmotic Pressure

Temperature, pH, and the osmotic pressure of the enzyme solution are also important factors affecting protoplast release. Temperature exerts a dual regulatory effect on protoplast preparation. Studies have shown that within a certain range, a gradual increase in temperature generally leads to a gradual rise in the number of prepared protoplasts [13]. However, high temperatures may cause a slight decrease in enzyme activity, which in turn reduces protoplast yield and activity [13]. It can also increase the fluidity of the receptor cell membrane, which may lead to premature lysis of protoplasts during centrifugation, resulting in their destruction. To avoid thermal damage, the incubation temperature for protoplast preparation should not exceed 35 °C, and most plant protoplasts can be stably isolated at room temperature (23–28 °C) [88,96]. Consistent with this principle, 28 °C is the optimal temperature for the enzymatic digestion of *G*. *biloba* calli for protoplast production [11]. The pH of the enzymatic hydrolysis solution can indirectly affect digestion efficiency by regulating the activity of cell wall-degrading enzymes [97]. Mannitol is a commonly used osmotic pressure regulator, and the optimal mannitol concentration for maintaining plant cell osmotic pressure in general studies ranges from 0.4 to 0.6 M [12]. Notably, higher osmotic pressure induces lower protoplast isolation efficiency, while lower osmotic pressure leads to more protoplast rupture [67]. Meanwhile, osmotic concentration directly affects the activity of cell wall-degrading enzymes. Protoplast isolation from *C. sativa* leaf has demonstrated that pectolyase performs significantly better in 0.4 M mannitol than in 0.7 M mannitol [40].

#### 4.2.5. Protoplast Purification Methods

Protoplast purification primarily focuses on removing large debris, such as cell tissues and undigested cell clusters, that affect the physiological state of protoplasts. Protoplast purification relies on various factors, such as nylon membrane pore size, centrifugation speed, and sucrose gradient centrifugation [56]. Protoplasts can be easily separated from incompletely digested tissues and harvested by adjusting the sieve pore size [88]. After enzymatic hydrolysis, the crude protoplast extract has a complex composition, potentially containing undigested tissues, cell clusters, and debris. It is commonly filtered through a 40–400 mesh cell sieve to obtain high-purity protoplasts [57]. Comparative studies have confirmed that pore sizes ranging from 20 μm to 88 μm differently affect the integrity of *L*. *corniculatus* leaf protoplasts, indicating that filtration parameters should be tailored to material characteristics [70]. Specifically, 44 μm and 62 μm nylon sieves yield higher protoplast integrity than 20 μm and 88 μm sieves [70]. Centrifugal speed exhibits a bidirectional effect: excessively high centrifugal force exerts strong pressure on protoplasts, easily causing severe damage; conversely, insufficient centrifugal force prevents complete collection of protoplasts [73]. Protoplast purification should be performed under gentle and low-speed centrifugation conditions, with particular care to avoid excessive centrifugal forces [56]. The gradient speed screening experiment evaluated 700 rpm as the optimal centrifugation parameter for protoplast isolation from *R*. *communis* leaf, achieving the maximum protoplast vitality (80.34%) [73]. Sucrose gradient centrifugation purifies viable protoplasts by removing cell wall debris and dead cells, and is a commonly used method for protoplast purification [98]. In sucrose gradient purification, sucrose concentration directly affects the banding and recovery of intact protoplasts. A 25% sucrose solution was confirmed optimal for purifying *C*. *morifolium* ‘White ND’ leaf protoplasts [42].

### 4.3. Brief Summary

Intrinsic variables, including the species of medicinal plants, the growth state of plant materials, and tissues of plant materials, and process variables, including pretreatment methods before enzymatic hydrolysis, the composition of the enzymatic hydrolysis solution, enzymatic hydrolysis time, rotation speed during enzymatic hydrolysis, external environmental factors, and the adopted protoplast purification method, can affect the efficiency of the enzymatic hydrolysis process, and further influence the yield and activity of the prepared protoplasts (Table 1). Cellulase combined with Macerozyme represents the most widely used enzyme combination in published research. Mechanical cutting, as the most frequently adopted pretreatment approach, is applicable to various medicinal plant species and explant types, delivering highly repeatable experimental results. Compared to herbaceous medicinal plants, woody medicinal plants typically require additional pectinase to enhance cell wall degradation efficiency due to their higher degree of lignification. Leaf is the most commonly selected explant material for protoplast isolation in herbaceous medicinal plants. Based on multiple studies, pretreatment methods, enzyme proportions, and mannitol concentration are the most significant process variables affecting protoplast preparation efficiency.

The common biological principles supporting successful protoplast isolation are as follows: through the coordinated regulation of pretreatment methods, enzymatic digestion parameters, and external osmotic conditions, cell walls derived from different species and tissues are specifically degraded at a suitable enzymatic digestion efficiency, with the integrity of the cell membrane structure and cellular physiological activity maintained to the maximum extent.

Based on the systematic comparison of intrinsic material traits and operational processing parameters, several practical design guidelines are concluded for protoplast preparation. Intrinsic variables, including medicinal plant species, explant types, and plant growth status, directly determine protoplast yield and viability. Young leaves and friable calli serve as the dominant materials for protoplast isolation, and herbaceous medicinal plants have been far more extensively studied than recalcitrant woody species. Process variables such as pretreatment, enzyme, time, rotation speed, temperature, pH, osmotic pressure, and protoplast purification methods exert dual effects on protoplasts, each with a corresponding optimal level. Moreover, the ideal isolation system varies greatly among different medicinal plant species.

## 5. Applications of Protoplasts

**Figure 2:**
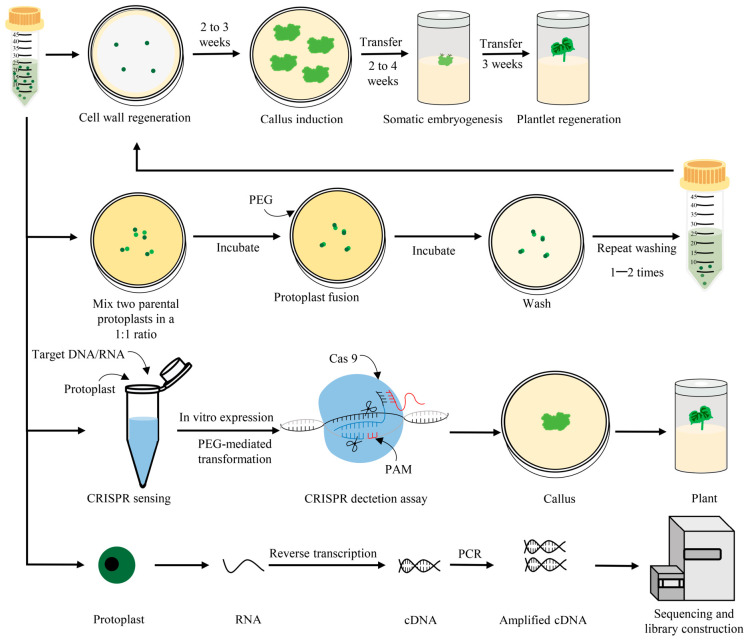
Schematic workflow of the main applications of protoplasts from medicinal plants. Purified protoplasts from medicinal plants have been widely applied in four major aspects: first, protoplast culture and regeneration research, which utilizes the totipotency of protoplasts to significantly shorten the cycle of breeding material creation; second, protoplast fusion technology, which breaks the barrier of interspecific hybridization incompatibility and creates somatic hybrids with the advantages of both parents; third, CRISPR/Cas9-mediated gene editing technology, which, combined with the protoplast culture and regeneration system, enables the acquisition of edited plants in a relatively short time; fourth, single-cell sequencing, which provides key technical support for germplasm resource identification, precision breeding, and gene function research.

Due to the absence of a cell wall, medicinal plant protoplasts can easily integrate exogenous genetic material, facilitating plant genetic transformation and holding great application prospects for gene function verification, somatic cell genetics, cell regulation, crop improvement, and breeding (Figure 2).

### 5.1. Transient Expression of Medicinal Plant Protoplasts

#### 5.1.1. Methods for Transient Expression of Medicinal Plant Protoplasts

**Figure 3:**
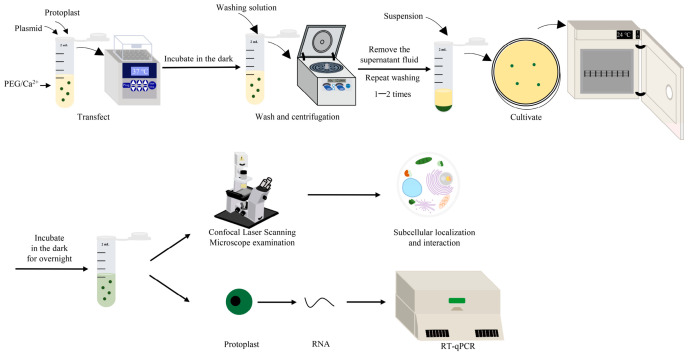
PEG-mediated transient transformation of the medicinal plant protoplasts and its main applications. The common operation of PEG-mediated transient transformation of the medicinal plant protoplasts is as follows: purified protoplasts and plasmids are added to PEG/Ca^2+^ buffer, followed by light-protected heat shock in a metal bath; the mixed solution is then washed and centrifuged, resuspended in solution, and incubated overnight in the dark. The protoplast transient expression system can not only be used for protein subcellular localization and protein–protein interaction detection to determine the high-expression region of fusion proteins and realize the regulation of cellular processes, but also for transient overexpression technology to achieve high-level gene expression in a short period of time.

Plant protoplasts are naked plant cells lacking a rigid cell wall and are well-known as efficient transformation tools in botanical research [27]. Protoplasts are widely used for genetic transformation due to their ability to dedifferentiate, proliferate, regenerate, and adsorb exogenous DNA [41]. Protoplast transient expression systems play a vital role in medicinal plant genetic improvement, gene function verification, and secondary metabolism research [115]. Various transient expression systems for medicinal plant protoplasts have been established, including the most commonly used PEG-mediated method and electroporation [87]. PEG-mediated method refers to the exogenous DNA that interferes with the surface potential of the cell membrane phospholipid bilayer through PEG, and enters the protoplast recipient cell [12] (Figure 3). To date, the PEG method has been mostly used in transient plasmid transformation of medicinal plant protoplasts, such as *Taxus*, *S*. *miltiorrhiza*, *A*. *catechu*, *U*. *rhynchophylla*, *E*. *ulmoides*, *P*. *ternata*, *G*. *biloba*, *A*. *senticosus*, *P*. *kurrooa*, *P*. *ginseng*, *F*. *esculentum*, *C*. *tinctorius*, and *C*. *sativa* [11,13,14,15,24,38,40,41,51,59,62,64,76,77,78,100,104,110,115]. Electroporation is a widely used physical transfection method in molecular and cell biology, which delivers exogenous DNA into protoplasts by placing them in a high electric field, low capacitance electric shock environment [12,116]. However, the reversible pores generated by electroporation may be difficult to self-repair in some cell membranes, leading to cell death due to content leakage [12]. A study employed electroporation technology to introduce the *CrWRKY1* promoter/GUS plasmid into protoplasts of *C*. *roseus* cell suspension cultures and monitored the activity of its promoter fragments [117]. PEG-mediated transient transfection is a commonly used method for genetic manipulation of medicinal plant protoplasts, but this method is species dependent, and the characteristics of the technology itself also impose certain constraints on its downstream application scenarios, such as gene editing and cell regeneration.

#### 5.1.2. Applications of Transient Expression in Promoter Analysis

Transient gene overexpression technology is suitable for plant protoplasts lacking stable genetic transformation systems, advancing research on gene function [12]. At present, several reporter genes, such as GUS, LUC, and GFP, have been successfully introduced into plant protoplasts [118]. Protoplast transient expression systems provide a physiological context for predicting promoter [119]. Multiple representative studies have fully demonstrated the versatility and effectiveness of this system. Specifically, the activation mechanism of the VRE1-CaMV35S minimal promoter was elucidated in *Euphorbia pulcherrima* protoplasts, confirming its dependence on VIP1 nuclear localization [120]. The crucial promoter regions for activity of the *CrWRKY1* promoter were defined via 5′ and 3′ terminal deletion analysis using *C*. *roseus* protoplasts [117]. The activities of four *C*. *tinctorius* flavonoid biosynthesis-related promoters (*pctCHI-1*, *pctCHS-2*, *pctF3H-2*, and *pctGT-1*) were characterized in safflower using a protoplast expression system [110]. Additionally, the function of the auxin response element DR5 synthetic promoter was verified in *C*. *sativa* protoplasts by flow cytometry [64]. Therefore, medicinal plant protoplast transient expression systems not only provide an efficient platform for deciphering gene expression regulatory mechanisms but also reveal the promoter functions.

#### 5.1.3. Applications of Transient Expression in Gene Identification

Due to their lack of a cell wall, protoplasts serve as ideal hosts for efficient co-transfection of multiple exogenous DNAs [56]. This technology has been widely applied in the functional exploration of the genes of various medicinal plants. Subcellular localization, protein–protein interaction, and transient overexpression experiments using *G*. *biloba* protoplasts enabled systematic analysis of gene functions and their regulatory networks [12]. Subcellular localization experiments confirmed the specific localization to the nucleus of the transcription factor UrWRKY37 in *U*. *rhynchophylla* protoplasts [13]. Transient expression in *R*. *communis* mesophyll protoplasts was used to elucidate the biochemical characteristics of RcFATA protein, which is a fatty acyl–acyl carrier thioesterase [73]. The functional roles of *BIS1*, *CrMYC2,* and *CrJAZ1* were analyzed using transient gene expression assays in *C*. *roseus* protoplasts [121]. Additionally, transient overexpression of CmMBF1c in protoplasts of the waterlogging-sensitive *C*. *morifolium* cultivar ‘Qinglu’ revealed the molecular mechanism by which CmMBF1c improves waterlogging tolerance by reducing ROS accumulation [122]. The combination of protoplast transient expression and BiFC technology is commonly used for protein interaction studies in medicinal plant protoplasts [56,123]. To date, this technology has been widely applied to the functional characterization of regulatory proteins and the activity analysis of metabolic enzymes in both woody and herbaceous medicinal plants, covering species such as *G*. *biloba*, *C*. *roseus*, and *C*. *tinctorius* [11,12,121,124]. Plant protoplasts can serve as a transient expression system to rapidly analyze the target genes of transcription factors, acting as an efficient experimental model for studying transcription factor activity.

### 5.2. Gene Editing Research

Protoplast transient transfection technology is widely used in gene silencing and genome editing research due to its high-efficiency screening characteristics [87]. Protoplasts combined with newly developed technologies, such as RNAi and CRISPR/Cas9, can be used for gene silencing and editing in different plant species [39]. RNAi technology represents an effective paradigm for elucidating plant stress resistance mechanisms. In *C*. *morifolium*, comparative analysis between waterlogging-sensitive and tolerant cultivars revealed that CmMBF1c positively regulates waterlogging tolerance by reducing ROS accumulation and enhancing ROS scavenging gene expression [122]. At present, protoplasts mainly use the CRISPR/Cas9-mediated gene editing system to evaluate the efficiency of genome editing mutagenesis, and this technology has been applied in various medicinal plants, such as *S*. *miltiorrhiza*, *C*. *sativa*, *Dioscorea* spp., and *Ganoderma lucidum* [24,50,51,125,126]. In addition to CRISPR/Cas9, the programmable nuclease TnpB is substantially smaller than Cas9 and has been successfully applied for gene editing in diverse medicinal plants, including *Artemisia annua*, *S*. *miltiorrhiza*, *Scutellaria baicalensis*, *Isatis indigotica*, and *C*. *pilosula*, holding great promise for molecular breeding to enhance crop yield and quality [127]. In recent years, protoplast-mediated genome editing has achieved substantial advances, including DNA-free genome editing, Cas12 systems, base editing, prime editing, ribonucleoprotein delivery, and multiplex editing, greatly expanding the precision editing toolbox for medicinal plants [128,129,130,131,132,133]. Therefore, combined with emerging gene editing technologies, medicinal plant protoplasts can be used for rapid silencing of target genes, providing an efficient method for studying the functions of key genes in secondary metabolic pathways and facilitating precise regulation and improvement of medicinal active ingredient synthesis.

### 5.3. Single-Cell Sequencing

Single-cell sequencing technologies, including scDNA-seq, scRNA-seq, and single-cell epigenomic sequencing, can construct high-dimensional maps of individual cells in isolated samples [134,135]. The presence of a cell wall makes plant single-cell isolation more challenging than that of an animal cell, resulting in a relatively limited scale of application of single-cell sequencing in plants [136]. The preparation of protoplasts is a necessary step for single-cell sequencing of medicinal plants. Using single-cell sequencing technology, distinct gene expression patterns of *C*. *roseus*, *G*. *pentaphyllum*, *H*. *perforatum*, *D*. *longan*, and *Taxus wallichiana* var. *mairei* were revealed within heterogeneous cell populations [36,65,71,107,108,137]. scRNA-seq technology overcomes the limitations of traditional bulk sequencing, enabling high-resolution analysis of cell heterogeneity, providing single-cell-level molecular regulatory mechanisms for precision breeding and metabolic engineering [138]. Conventional protoplast-based scRNA-seq technology faces two major challenges: digestion-induced transcriptional artifacts and the loss of fragile cell types [138,139]. To overcome this limitation, snRNA-seq has emerged as a highly promising alternative that bypasses the requirement for protoplast isolation and effectively circumvents transcriptional perturbations arising from enzymatic digestion [140]. In addition, the integration of single-cell transcriptomics with spatial transcriptomics effectively compensates for the spatial information loss inherent in cell dissociation, enabling precise spatial localization of cell subpopulations and a deeper understanding of their physiological functions [141].

### 5.4. Cell Fusion

Protoplasts are important raw materials for plant cell fusion technology (also called somatic hybridization), which can break through reproductive isolation between distant species, achieve gene recombination, and create new varieties with excellent traits from two species [56]. Protoplast fusion technology enables asexual transfer of genetic material, facilitating the renewal of germplasm resources and improving breeding efficiency. A standard cell fusion strategy typically includes four key steps: isolating parental protoplasts, inducing protoplast fusion through chemical (PEG-mediated) or electrical stimulation, culturing heterokaryons to regenerate hybrid calli, and screening target somatic hybrid lines [142]. Compared with model plants, research on protoplast fusion in medicinal plants is relatively limited, mainly through chemical PEG stimulation of protoplast fusion, such as *Kitagawia terebinthacea*/*Bupleurum chinense*, *D*. *candidum*/*G*. *pentaphyllum*, *Z*. *officinale* ‘Lushan Zhangliang jiang’/*Z*. *officinale* ‘Chenggu Huang Jiang’, *B*. *scorzonerifolium*/*Taxus chinensis* var. *mairei* and *J*. *sambac*/*J*. *mesnyi* [72,101,143,144,145]. Protoplast cell fusion technology enhances the construction of efficient metabolic systems, offering revolutionary prospects for germplasm innovation, seed propagation, and crop variety improvement.

### 5.5. Plant Regeneration

A protoplast, an isolated naked plant cell, can regenerate a cell wall, undergo dedifferentiation and mitosis to form calli, and further develop an adventitious shoot and root or somatic embryo, ultimately regenerating a whole plant [88,100,146]. By evaluating a large number of protoplast-regenerated plants, it is expected to screen mutants with ideal traits [147]. Compared with model plants and cash crops, most investigations on medicinal plant protoplasts are merely limited to cell wall regeneration, while systems capable of whole plant regeneration remain scarce. Several representative documented advances are summarized as follows: *C*. *sativus*, *P*. *ternata*, *Rh*. *sachalinensis*, *C*. *morifolium* ‘White ND’, *A*. *gigas*, *S*. *miltiorrhiza*, *P*. *amurense*, *Z*. *officinale*, *C*. *pilosula*, *P*. *cordata*, *A*. *melilotoides*, *S*. *dulcamara*, *E*. *cardamomum*, *L*. *usitatissimum*, *L*. *corniculatus*, *S*. *album*, *S*. *nigrum*, *P ginseng*, *B*. *scorzonerifolium*, *P*. *praeruptorum*, *S*. *xanthocarpum*, *Rehmannia glutinosa*, *C*. *roseus*, and *L*. *barbarum* [18,22,25,42,43,50,67,68,70,74,75,78,79,80,81,83,102,103,106,109,111,112,113,114,148,149,150].

The regeneration efficiency of plant protoplasts is constrained by both intrinsic and extrinsic factors. Genotype effects cause marked differences in dedifferentiation and redifferentiation capacity across species and tissue sources, with most recalcitrant species failing to regenerate whole plants [151]. Hormonal regulation is limited by the poor stability of natural hormones and the species-specific hormonal requirements, hindering the generalizability of regeneration systems [57]. Epigenetic barriers suppress the expression of key regeneration genes, leading to abnormal regeneration [152]. Oxidative stress during protoplast isolation leads to excessive ROS accumulation [153]. Without timely antioxidant systems, protoplasts suffer oxidative damage, resulting in cellular structural disruption and cell division, which hinder plant regeneration [153]. Furthermore, the overaccumulation of ROS triggers somaclonal variation, resulting in unstable regeneration efficiency, abnormal phenotypes, and metabolic disorders [154].

Whole plant regeneration of protoplasts derived from most medicinal plants currently relies on the indirect organogenesis pathway of “calli-adventitious shoot and root-regenerated plant”, laying a material foundation for germplasm innovation and efficient production of bioactive metabolites.

## 6. Conclusions

The medicinal plant protoplast transformation system has important roles in rapid, high-throughput, and large-scale deciphering of promoter and gene functions. However, overall, the number of fully established medicinal plant protoplast preparation and transformation systems is much smaller than that of model plants and crops. To date, protoplast isolation systems have been successfully established for a wide range of medicinal plant species, mainly herbaceous plants.

Plant materials are the foundation for isolating protoplasts from medicinal plants. Before isolating medicinal plant protoplasts via enzymatic hydrolysis, pretreatment methods, such as vacuum treatment, dark treatment, pre-plasmolysis treatment, low-temperature treatment, and mechanical cutting, can be applied to increase protoplast yield and purity. For the purification of medicinal plant protoplasts, multiple factors, such as sieve pore size, centrifugation speed, and sucrose concentration, should be considered. Application of the medicinal plant protoplast transient transfection system enables rapid detection of promoter activity, elucidation of signal transduction pathways, identification of gene functions, and verification of the regulatory network. Although gene function verification systems have been established, medicinal plant protoplasts also serve as ideal recipients for gene silencing and gene editing technologies. In addition, the single-cell characteristics of protoplasts can be utilized to analyze the molecular regulatory mechanisms in medicinal plants. At present, the regeneration of whole medicinal plants starting from protoplasts mostly takes callus formation as an intermediate step, followed by the gradual differentiation of adventitious buds and roots, and ultimately developing into a complete plant individual.

Protoplast technology can effectively overcome the inherent limitations of medicinal plants. However, it still faces numerous bottlenecks. Due to genotype dependence, medicinal plants generally exhibit species and tissue specificity, making it difficult to establish efficient and stable systems for protoplast preparation, purification, and transient expression. Existing research on gene editing, single-cell sequencing, and protoplast fusion remains largely limited to a few species. The scalability for large-scale breeding programs is insufficient. In addition, most protoplast fusion experiments are conducted on model plants such as tobacco. Most regeneration studies only achieve cell wall reconstruction and callus induction, and whole-plant regeneration from protoplasts suffers from poor controllability. In addition, there is a lack of standardized protocols among different research groups regarding enzyme batches, physiological states of plant materials, and culture conditions, resulting in poor reproducibility across laboratories.

Protoplast biotechnology has shown considerable economic potential in medicinal plant breeding and metabolic engineering. Subsequently, economic competitiveness should focus on enzyme recycling, domestic equipment substitution, and synthetic biology-driven optimization. To promote medicinal plant protoplast technology from fundamental laboratory research toward industrial application and large-scale breeding programs, future studies can focus on the following four directions. First, AI-assisted parameter optimization remains a long-term prospective hypothesis for protoplast isolation that, despite rapid advances in enzyme engineering, awaits empirical validation, with future potential to predict optimal conditions by integrating multidimensional constraints. Second, the delivery protocol of CRISPR RNP complexes into protoplasts should be refined through material modification and fine-tuning of experimental conditions, so as to improve the efficiency and biosafety of transgene-free gene editing. Third, automated workflow platforms and high-throughput screening systems should be integrated with microfluidic devices or automated equipment to improve experimental stability and adaptability for large-scale breeding. Fourth, protoplast technology should be deeply integrated with spatial transcriptomics and multi-omics to dissect the regulatory networks and cellular heterogeneity of secondary metabolic pathways in medicinal plants at the single-cell level. Through interdisciplinary integration of AI, multi-omics analysis, and synthetic biology, a comprehensive system covering upstream material preparation and downstream experimental applications can be established. This system provides strong technical support for new variety breeding, targeted genetic improvement, metabolic engineering of bioactive products, and ecological conservation breeding.

## Figures and Tables

**Table 1:** Intrinsic and process variables affecting the preparation of medicinal plant protoplasts.

Intrinsic Variables			Process Variables						
Specie	Tissue	Reference	Enzyme	Pretreatment	Temperature/°C	Mannitol Concentration	Time/h	Rotation Speed	Purification Method
*G*. *biloba*	Callus	[11]	3 % Cel-R10 0.25 % Pec-Y23	NR	28	0.3 M	3	NR	Cen
*G*. *biloba*	Leaf	[12]	2% Cel 0.25% Pec	MC	28	0.4 M	3	1× *g*	Cen
*U*. *rhynchophylla*	Leaf	[13]	1.25% Cel-R10 0.6% Mac-R10	MC + Plas	26	0.8 M	5	40 rpm/min	Cen
*E*. *ulmoides*	Stem	[14]	2.5% Cel-R10 0.6% Mac-R10 2.5% Pec 0.5% Hemi	MC + Plas + Dark + Vac	25	0.6 M	10	40–50 rpm/min	Cen
*Taxus*	Calli	[15]	1.0% Cel-R10 0.5% Mac-R10 0.15% Pec-Y23	MC + Vac	RT	0.5 M	2	NR	Cen
*Taxus*	Leaf	[15]	1.0% Cel-R10 0.5% Mac-R10 0.15% Pec-Y23	MC + Vac	RT	0.5 M	2	NR	Cen
*B*. *striata*	Leaf	[16]	1.5% Cel-R10 0.4% Pec-Y23 0.5% Mac-R10	MC + Dark + LT + Plas	CT	0.75 M	4	120 rpm/min	Cen
*C*. *sativus*	Calli	[18]	0.1% Pec-Y23 1% Cel-R10 1% Drise	NR	NR	0.3 M	2.5–3	NR	Cen
*M*. *cordata*	Leaf	[19]	0.2% Pec-Y23 0.5% Cel-R10 0.1% Mac-R10	MC + Plas	30	0.6 M	6	NR	Cen
*I*. *rotunda*	Leaf	[20]	3% Cel-R10 0.4% Mac-R10	MC	40	0.7 M	6	50 rpm/min	Cen
*Rh*. *sachalinensis*	Leaf	[22]	1.0% Cel-R10 0.5% Mac-R10	MC	25	0.5 M	4	80 rpm/min	Cen
*S*. *miltiorrhiza*	Leaf	[24]	1.5% Cel-R10 0.4% Mac-R10	MC	RT	0.4 M	4–5	40 rpm	Cen
*A*. *gigas*	Leaf	[25]	1.0% Vz-L 1% Cc-1.5L 0.5% Px-XXL	NR	24 ± 1	0.4 M	7	40 rpm	Cen
*C*. *roseus*	Leaf	[36]	1% Cel-R10 0.15% Mac-R10	MC	RT	0.45 M	2	NR	Cen
*A*. *catechu*	Leaf	[38]	2% Cel-R10 0.5% Mac-R10	MC	25	0.7 M	12	40 rpm	Cen
*R*. *communis*	Leaf	[39]	1.5% Cel-R10 0.4% Mac-R10	MC	26	0.4 M	3	50 rpm	Cen
*R*. *communis*	Cotyledon	[39]	1.5% Cel-R10 0.4% Mac-R10	MC	26	0.4 M	9	50 rpm	Cen
*C*. *sativa*	Leaf	[40]	1.5% Cel-R10 0.4% Mac-R10 1.0% Pec-Y23	MC + Vac	23	0.4 M	15	50–70 rpm	Flo
*C*. *morifolium* ‘White ND’	Leaf	[42]	1.5% Cel-R10 0.3% Mac-R10 0.1% Drise	MC + Plas	25 ± 2	0.5 M	4	40 rpm	Int
*P*. *ternata*	Cell suspension	[43]	2% Cel-R10 10% Pec 0.01% Pec-Y23	NR	25 ± 1	NR	5–20	30 rpm	Cen
*N*. *tenuifolia*	Leaf	[52]	Cel-R10 Mac-R10	MC	NR	8%	NR	NR	NR
*C*. *oleifera*	Leaf	[58]	1.5% Cel-R10 0.5% Mac-R10 0.25% Sna	MC + Vac	28	0.4 M	14	40 rpm/min	Cen
*A*. *senticosus*	Callus	[59]	1.5% Cel 0.2% Mac	NR	RT	0.6 M	6	50 rpm	Cen
*C*. *sativa*	Leaf	[62]	2.5% Cel-R10 0.3% Mac-R10	MC	28	0.7 M	16	WA	Cen
*C*. *roseus*	Leaf	[63]	2% Cel-R10 0.3% Mac-R10 0.1% Pec	MC + Vac	25	0.4 M	3	NR	Cen
*C*. *sativa*	Leaf	[64]	1.25% Cel-R10 0.3% Mac-R10 0.075% Pec-Y23	MC	RT	0.4 M	16	75 rpm	Cen
*C*. *roseus*	Leaf	[65]	2% Cel-R10 0.3% Mac-R10 0.1% Pec	MC + Vac	RT	0.45 M	2.5	NR	Cen
*C*. *oleifera*	Petal	[66]	3% Cel-R10 1% Mac-R10	NR	RT	0.5 M	8	NR	Cen
*P*. *amurense*	Leaf	[67]	1% Cel-R10 1% Drise-20	MC	25 ± 1	0.6 M	6	NR	Cen
*E*. *cardamomum*	Leaf	[68]	0.5% Mac-R10 2% Cel-R10	MC	25	9%	18–20	WA	Cen
*E*. *cardamomum*	Cell suspension	[68]	1% Mac-R10 2% Cel-R10	WP	25	8%	24	53 rpm	Cen
*Z*. *officinale*	Leaf	[68]	0.5% Mac-R10 3% Hemi 5% Cel-R10	MC	15 (10 h) + 30 (6 h)	7%	16	53 rpm	Cen
*Z*. *officinale*	Cell suspension	[68]	1% Mac-R10 3% Hemi 6% Cel-R10	WP	15 (10 h) + 30 (8 h)	7%	18	53 rpm	Cen
*C*. *morifolium* ‘Crystal Regal’ + ‘ZihongTuogui’ + ‘Zi Fengche’	Petal	[69]	10 g/L Cel 6 g/L Mac 2 g/L Drise	MC + Plas + Dark	22	0.4 M	12–14	30 rpm/min	Cen
*C*. *morifolium* ‘Crystal Regal’ + ‘ZihongTuogui’ + ‘Zi Fengche’	Leaf	[69]	10 g/L Cel 6 g/L Mac 2 g/L Drise	MC + Plas + Dark	22	0.4 M	12–14	30 rpm/min	Cen
*L*. *corniculatus*	Leaf	[70]	2% Cel-R10 0.5% Mac-R10 0.1% Pec-Y23	MC + Plas	28	13%	16	WA	Cen
*L*. *corniculatus*	Cotyledon	[70]	2% Cel-R10 1% Macs 0.1% Pec-Y23	MC + Plas	28	13%	6–16	50 rpm	Cen
*G*. *pentaphyllum*	Leaf	[71]	1% Cel-R10 0.15% Mac-R10 0.1% Pec	MC	25	0.45 M	3–3.5	NR	Cen
*D*. *candidum*	Leaf	[72]	1.5% Cel-R10 1% Pec-R10	NR	25 ± 1	0.5 M	4–6	5 rpm/min	Int
*G*. *pentaphyllum*	Leaf	[72]	2% Cel-R10 1.5% Pec-R10	NR	25 ± 1	0.5 M	4–6	5 rpm/min	Int
*R*. *communis*	Leaf	[73]	1.6% Cel-RS 0.8% Mac-R10	MC + Plas + Dark	25	0.5 M	4/3	50 rpm	Cen
*C*. *pilosula*	Callus	[74]	1.5% Cel-R10 3% Pec	NR	26 ± 1	0.4 M	3	70 rpm/min	Flo
*P*. *cordata*	Leaf	[75]	1% Cel 1% Mac	LT + Dark + MC	25	130 g/L	4	50–60 rpm/min	Cen
*E*. *ulmoides*	Callus	[76]	1.5% Cel-R10 1.0% Mac-R10	MC	25	0.6 M	4	70 rpm	Cen
*P*. *kurrooa*	Leaf	[77]	2% Cel-R10 0.6% Mac-R10	MC + Plas	25	0.6 M	4	50 rpm	Cen
*C*. *sativa*	Leaf	[78]	0.5% Cel-R10 0.05% Pec-Y23	MC + Plas + Dark	26	0.5 M	16	35 rpm	Cen
*C*. *sativa*	Callus	[79]	1.25% Cel-R10 0.3% Mac-R10 0.075% Pec-Y23	MC + Plas	25	0.4 M	16	75 rpm/min	Cen
*Z*. *officinale*	Cell suspension	[80]	4% Cel-R10 1% Mac-R10 0.1% Pec-Y23	NR	27	11%	12–14	NR	Cen
*T*. *canadensis*	Cell suspension	[82]	1% Cel 0.1% Pec-Y23	NR	23–25	0.5 M	4	120 rpm/min	NR
*S*. *dulcamara*	Cell suspension	[83]	2% Rhz-HP150 2% Mei 0.03% Mac-R10	NR	25	13%	16	440 rpm	Flo
*Erigeron breviscapus*	Cell suspension	[99]	1% Cel 0.3% Pec 0.5% Hemi	NR	25	NR	5	60 rpm/min	Cen
*E*. *breviscapus*	Leaf	[99]	0.5%Cel 0.2% Pec 0.5% Mac	MC	25	NR	12	60 rpm/min	Cen
*P*. *ternata*	Leaf	[100]	20 g/L Cel-R10 15 g/L Mac-R10	MC + Vac	RT	0.4 mM	6	NR	Cen
*Jasminum sambac* L. + *Jasminum mesnyi*	Leaf	[101]	1.5% Cel-R10 0.4% Mac-R10 0.8% Pec	MC + Vac	26–30	0.4 M	8	50 rpm	Int
*J*. *sambac* + *J*. *mesnyi*	Callus	[101]	1.5% Cel-R10 0.4% Mac-R10 0.8% Pec	MC + Vac	26	0.4 M	4	50 rpm	Int
*J*. *sambac* + *J*. *mesnyi*	Petal	[101]	1.5% Cel-R10 0.4% Mac-R10 0.8% Pec	MC + Vac	26–30	0.4 M	10	50 rpm	Int
*Linum usitatissimum* L. (Flax)	Root	[102]	2% Rhz-HP150 4% Mei 0.3% Mac	MC + Plas	25	13%	16	30–40 rpm	Int
*L*. *usitatissimum*	Hypocotyl	[102]	2% Rhz-HP150 4% Mei 0.3% Mac	MC + Plas	25	13%	16	30–40 rpm	Flo
*L*. *usitatissimum*	Cotyledon	[102]	2% Rhz-HP150 4% Mei 0.3% Mac	MC + Plas	25	13%	16	30–40 rpm	Cen
*L*. *corniculatus*	Root	[103]	0.8% Cel-R10 0.4% Drise 0.8% Rhz-HP150 0.4% Mac-R10	MC + Plas	28	13%	6–16	WAA	Cen
*L*. *corniculatus*	Hypocotyl	[103]	0.8% Cel-R10 0.4% Drise 0.8% Rhz-HP150 0.4% Mac-R10	MC + Plas	28	13%	6–16	WA	Cen
*L*. *corniculatus*	Cotyledon	[103]	0.8% Cel-R10 0.4% Drise 0.8% Rhz-HP150 0.4% Mac-R10	MC + Plas	28	13%	6–16	WA	Cen
*L*. *corniculatus*	Leaf	[103]	0.5% Cel-R10 0.5% Rhz-HP150 0.25% Mac-R10	Plas	28	13%	16	WA	Cen
*L*. *corniculatus*	Cell suspension	[103]	4% Mei 2% Rhz-HP150 0.3% Mac-R10	NR	28	13%	16	80 rpm	Cen
*Fagopyrum esculentum*	Hypocotyl	[104]	1.0% Cel-RS 0.25% Mac-R10 0.02% Pec-Y23	MC + Vac	25	0.4 M	2	80 rpm	Cen
*F*. *esculentum*	Cotyledon	[104]	1.0% Cel-RS 0.25% Mac-R10 0.02% Pec-Y23	MC + Vac	25	0.4 M	2	80 rpm	Cen
*Taxus yunnanensis*	Callus	[105]	2.0% Cel-R10 1.0% Pec	NR	25 ± 2	0.45 M	10–12	WS	Cen
*Santalum album* L.	Cell suspension	[106]	1.0% Cel-RS 1.0% Mac-R10 0.5% Drise	NR	25	0.55 M	4–5	1.5 rpm	Flo
*Hypericum perforatum*	Leaf	[107]	1.25% Cel 0.3% Pec	MC	NR	0.4 M	NR	NR	Cen
*H*. *perforatum*	Petal	[107]	1.25% Cel 0.3% Pec	MC	NR	0.4 M	NR	NR	Cen
*Dimocarpus longan* Lour.	Cell suspension	[108]	1.5% Mac 1.5% Cel-R10	NR	25	12.7%	12	NR	Cen
*Solanum nigrum* L.	Leaf	[109]	0.7% Cel-EA3-867	MC	28	0.5 M	3	NR	Cen
*Carthamus tinctorius* L.	Petal	[110]	1.5% Cel-RS 0.75% Mac-R10	MC + Plas + Dark	NR	0.6 M	NR	NR	Cen
*Panax ginseng* C.A.Meyer (Araliaceae)	Callus	[111]	2% CelLys 1% Pec 1% Macs	Plas	25	12%	5–6	50 rpm (1 h) + 40 rpm (4–5 h)	Cen
*Bupleurum scorzonerifolium* Willd	Calli	[112]	1.5% Cel-R10 0.3% Mac-R10	NR	25	0.6 M	4	OS	Cen
*Peucedanum praeruptorum* Dunn	Calli	[113]	1.5% Cel-R10 0.3% Mac-R10 0.5% Hel	NR	25	0.6 M	5	NR	NR
*Solanum xanthocarpum*	Leaf	[114]	1.5% Cel-R10 0.5% Mac-R10	NR	25	0.25 M	6–8	NR	Flo

## Data Availability

No new data were created or analyzed in this study. Data sharing is not applicable to this article.

## Abbreviations

The following abbreviations are used in this manuscript:

GUS	β-glucuronidase
PEG	Polyethylene glycol
FDA	Fluorescein diacetate
CPW	Cell and Protoplast Washing Salt Solution
Cel	Cellulase
Pec-Y23	Pectolyase-Y23
Mac	Macerozyme/Dissociative enzyme
Pec	Pectinase
Hemi	Hemicellulase
Drise	Driselase
Vz-L	Viscozyme^®^L
Cc-1.5L	Celluclast^®^1.5L
Px-XXL	Pectinex^®^XXL
Macs	Macerase
Sna	Snailase
Rhz-HP150	Rhozyme-HP150
Mei	Meicelase
CelLys	Cellulysin
Hel	Helicase
MC	Mechanical cutting
Plas	Plasmolysis
LT	Low-temperature
NR	Not reported
Vac	Vacuum
WP	Without Pretreatment
CT	Constant temperature
RT	Room temperature
rpm	revolutions per minute
min	Minute
g	Standard gravity
WA	Without agitation
cycles/min	Cycles per minute
WS	Without shaking
OS	Occasional shaking.
Cen	Centrifugation
Flo	Flotation
Int	Interface
M	Molar
h	Hour
mM	Millimolar
MPa	Megapascal
*w*/*v*	Weight/Volume
pH	Potential of hydrogen
μm	Micrometer
DNA	DeoxyriboNucleic Acid
RNA	Ribonucleic Acid
CRISPR	Clustered regularly interspaced short palindromic repeats
Cas	Clustered regularly interspaced short palindromic repeats-associated protein
TnpB	Transposon-encoded protein B
PAM	Protospacer adjacent motif
cDNA	Complementary Deoxyribonucleic Acid
PCR	Polymerase chain reaction
RT-qPCR	Real-time quantitative polymerase chain reaction
LUC	Luciferase
GFP	Green fluorescent protein
ROS	Reactive oxygen species
BiFC	Bimolecular fluorescence complementation
scDNA-seq	Single-cell Deoxyribonucleic Acid sequencing
scRNA-seq	Single-cell Ribonucleic Acid sequencing
snRNA-seq	Single-nucleus Ribonucleic Acid sequencing
AI	artificial intelligence
RNP	ribonucleoprotein

## References

[1] Sagayaraj C., Kumar K.B., Vimal S., Danya U. (2025). Assessment of Phytochemical Profile, Antioxidant, and Anticancer Activity Against Colon Cancer-HT-29: A Potent Therapeutic Medicinal Plant (*Tarenna alpestris*) in Megamalai Hills, Western Ghats, India. Appl. Biochem. Biotech..

[2] Balunas M.J., Kinghorn A.D. (2005). Drug discovery from medicinal plants. Life Sci..

[3] Yu J., Bai M., Wang C., Wu H., Liang X. (2024). Regulation of secondary metabolites accumulation in medicinal plants by rhizospheric and endophytic microorganisms. Med. Plant Biol..

[4] Tmušić N., Ilić Z.S., Milenković L., Šunić L., Lalević D., Kevrešan Ž., Mastilović J., Stanojević L., Cvetković D. (2021). Shading of Medical Plants Affects the Phytochemical Quality of Herbal Extracts. Horticulturae.

[5] Wu Z., Hu Y., Hao R., Li R., Lu X., Itale M.W., Yuan Y., Zhu X., Zhang J., Wang L. (2025). Research Progress of Genomics Applications in Secondary Metabolites of Medicinal Plants: A Case Study in Safflower. Int. J. Mol. Sci..

[6] Cheng L., Wang Z., Zhu Q., Ye M., Ye C. (2025). A long road ahead to reliable and complete medicinal plant genomes. Nat. Commun..

[7] Wang Y., Liu Z., Zhao A., Su M., Xie G., Jia W. (2009). Functional genomic approaches to explore secondary metabolitesin medicinal plants. Chin. J. Chin. Mater. Med..

[8] Han Y., Sun T., Tang Y., Yang M., Gao W., Wang L., Sui C. (2025). Root rot in medicinal plants: A review of extensive research progress. Front. Plant Sci..

[9] Wu D., Wei T., Cao D., Zang R., Xu H., Guo P. (2021). Application of Plant Protoplast Culture in Medicinal Plants. Life Sci. Res..

[10] Reyna-Llorens I., Ferro-Costa M., Burgess S.J., Lunn J. (2023). Plant protoplasts in the age of synthetic biology. J. Exp. Bot..

[11] Le L., Xie X., Zhang W., Ma Y., Wang Y., Fu F., Wang G., Cao F., Yang X. (2025). Development and application of a *Ginkgo biloba* L. callus-derived protoplast transient expression system for exploring the roles of GbMYB11 and GbbHLH3 in flavonoid metabolism. Ind. Crops Prod..

[12] Han X., Rong H., Feng Y., Xin Y., Luan X., Zhou Q., Xu M., Xu L.-a. (2023). Protoplast isolation and transient transformation system for *Ginkgo biloba* L.. Front. Plant Sci..

[13] Shao Y., Mu D., Pan L., Wilson I.W., Zheng Y., Zhu L., Lu Z., Wan L., Fu J., Wei S. (2023). Optimization of Isolation and Transformation of Protoplasts from *Uncaria rhynchophylla* and Its Application to Transient Gene Expression Analysis. Int. J. Mol. Sci..

[14] Hu B., Dong M., Liu R., Shan W., Wang Y., Ding Y., Peng J., Meng L., Wang C., Zhou Q. (2024). Establishment of an Efficient Protoplast Isolation and Transfection Method for *Eucommia ulmoides* Oliver. Front. Biosci..

[15] Lu P., Ji J., Fan F., Liu T., Shi Z., Li W., Sun C. (2025). Genome-Wide Characterization of the *SnRK* Gene Family in *Taxus* and Homologous Validation of *TaSnRK1.2* as a Central Regulator in Stress-Responsive Transcriptional Networks. Plants.

[16] Xu D., Zhang L., Chu S., Wei X., Qian G., Zheng M. (2016). A Protocol for the Isolation and Purification of Protoplastfrom *Bletilla striata* Leaves. Bull. Bot. Res..

[17] Liu J., Liu J., Cheng Y., Zhong X., Chen Z. (2010). Acquiring homozygous tetraploid germplasm by PEG-mediated protoplast fusion of *Rhodiola sachalinensis*. Chin. J. Chin. Mater. Med..

[18] Chaloushi B., Zarghami R., Abd-Mishani C., Omidi M., Agayev Y.M., Sardo B.P. (2007). Effects of Different Hormonal Treatments on the Callus Production and Plantlet Regeneration in Saffron (*Crocus sativus* L.). Pak. J. Biol. Sci..

[19] Wang P., Huang P., Liu W., Zeng J., Rao L. (2020). Isolation and Purification of Protoplasts from Mesophyll Tissues of *Macleaya cordata*. Mol. Plant Breed..

[20] Xu D., Huang R., Liu X., Gu Y., Hu B.g, Du Q. (2023). The Establishment and Optimization for the Extraction Method of Protoplasts from *Ilex rotunda* Thunb-Leaves. Mol. Plant Breed..

[21] Cheng J., Chen Y., Guo F., Dong P., Zhou C., Liang W., Wang H. (2025). Regulatory mechanisms and biosynthesis of chlorogenic acid in *Lonicera japonica*: Insights from tissue culture and inducer treatments. Front. Plant Sci..

[22] Liu J., Cheng Y., Chen Z. (2009). Protoplast isolation and plant regeneration from leaves of *Rhodiola sachalinensis*. Chin. Tradit. Herb. Drugs.

[23] Yoo S.D., Cho Y.H., Sheen J. (2007). *Arabidopsis* mesophyll protoplasts: A versatile cell system for transient gene expression analysis. Nat. Protoc..

[24] Shao J., Peng B., Zhang Y., Yan X., Yao X., Hu X., Li L., Fu X., Zheng H., Tang K. (2024). A high-efficient protoplast transient system for screening gene editing elements in *Salvia miltiorrhiza*. Plant Cell Rep..

[25] Lee H.S., Han J.E., Jie E.Y., Kim S.W., Kwon H.J., Lee G.M., Lee H.S., Murthy H.N., Park S.Y. (2024). Isolation, culture of protoplasts of *Angelica gigas* Nakai and regeneration of plants via somatic embryogenesis. Plant Cell Tiss. Org..

[26] Zhu L., Wang B., Zhou J., Chen L., Dai C., Duan C. (2005). Protoplast isolation of callus in *Echinacea augustifolia*. Colloids Surf. B Biointerfaces.

[27] Davey M.R., Anthony P., Power J.B., Lowe K.C. (2005). Plant protoplasts: Status and biotechnological perspectives. Biotechnol. Adv..

[28] Klercker J. (1892). Eine methode zur isolier lebender protoplasten. Ofvers. Kongl. Vetensk.-Akad. Forh..

[29] Cocking E.C. (1960). A Method for the Isolation of Plant Protoplasts and Vacuoles. Nature.

[30] Nagata T., Takebe I. (1970). Cell wall regeneration and cell division in isolated tobacco mesophyll protoplasts. Planta.

[31] Nagata T., Takebe I. (1971). Plating of isolated tobacco mesophyll protoplasts on agar medium. Planta.

[32] Carlson P.S., Smith H.H., Dearing R.D. (1972). Parasexual interspecific plant hybridization. Proc. Natl. Acad. Sci. USA.

[33] Melchers G., Sacristán M.D., Holder A.A. (1978). Somatic hybrid plants of potato and tomato regenerated from fused protoplasts. Carlsberg Res. Commun..

[34] Gosch G., Bajaj Y.P.S., Reinert J. (1975). Isolation, culture, and induction of embryogenesis in protoplasts from cell-suspensions of *Atropa belladonna*. Protoplasma.

[35] Schieder O. (1975). Regeneration von haploiden und diploiden *Datura innoxia* Mill. Mesophyll-Protoplasten zu Pflanzen. Z. Pflanzenphysiol..

[36] Sun S., Shen X., Li Y., Li Y., Wang S., Li R., Zhang H., Shen G., Guo B., Wei J. (2023). Single-cell RNA sequencing provides a high-resolution roadmap for understanding the multicellular compartmentation of specialized metabolism. Nat. Plants.

[37] Du J.G., Zhang H.T., Li W.L., Li X.Y., Wang Z., Zhang Y., Xiong A.S., Li M.Y. (2023). Optimization of Protoplast Preparation System from Leaves and Establishment of a Transient Transformation System in *Apium graveolens*. Agronomy.

[38] Wang Y., Wang L., Liu H., Gou B., Hu W., Qin L., Shen W., Wang A., Cui H., Dai Z. (2023). Direct leaf-peeling method for areca protoplasts: A simple and efficient system for protoplast isolation and transformation in areca palm (*Areca catechu*). BMC Plant Biol..

[39] Bai L., Cheng Y., She J., He Z., Liu H., Zhang G., Cao R., Chen Y. (2020). Development of an efficient protoplast isolation and transfection system for castor bean (*Ricinus communis* L.). Plant Cell Tiss. Org..

[40] Kim A.L., Yun Y.J., Choi H.W., Hong C.-H., Shim H.J., Lee J.H., Kim Y.-C. (2022). Establishment of efficient *Cannabis* (*Cannabis sativa* L.) protoplast isolation and transient expression condition. Plant Biotechnol. Rep..

[41] Cao C., Wang W., Yang X., Li D. (2023). Isolation of Protoplast and Establishment of Transient Expression System in *Areca catechu*. Mol. Plant Breed..

[42] Adedeji O.S., Naing A.H., Kim C.K. (2020). Protoplast isolation and shoot regeneration from protoplast-derived calli of *Chrysanthemum* cv. White ND. Plant Cell Tiss. Org..

[43] He Y., Zhu C., He M., Hao S. (1996). Protoplast culture and plant regeneration of *Pinellia ternata*. Plant Cell Rep..

[44] Babiychuk E., Kushnir S., Gleba Y.Y. (1992). Spontaneous extensive chromosome elimination in somatic hybrids between somatically congruent species *Nicotiana tabacum* L. and *Atropa belladonna* L.. Theor. Appl. Genet..

[45] Xie H., Liu B., Zhang Z., Bao Z., Tao W., He M. (1996). Intergeneric Protoplast Fusion between *Nicotiana undulata* L. and *Lycium barbarum* L. and Regeneration of Hybrid Plantlets. Acta Agron. Sin..

[46] Krumbiegel G., Schieder O. (1979). Selection of somatic hybrids after fusion of protoplasts from *Datura innoxia* Mill. and *Atropa belladonna* L.. Planta.

[47] Kushnir S.G., Shlumukov L.R., Pogrebnyak N.J., Berger S., Gleba Y. (1987). Functional cybrid plants possessing a *Nicotiana* genome and an *Atropa* plastome. Mol. Gen. Genet..

[48] Kushnir S., Babiychuk E., Bannikova M., Momot V., Komarnitsky I., Cherep N., Gleba Y. (1991). Nucleo-cytoplasmic incompatibility in cybrid plants possessing an *Atropa* genome and a *Nicotiana* plastome. Molec. Gen. Genet..

[49] Xue Y., Hiti-Bandaralage J.C.A., Hu Z., Zhao Z., Mitter N. (2023). First Report on Mesophyll Protoplast Isolation and Regeneration System for the *Duboisia* Species. Plants.

[50] Hsu C.T., Chiu C.C., Hsiao P.Y., Lin C.Y., Cheng S., Lin Y.C., Yang Y.L., Wu F.H., Harn H.J., Lin S.Z. (2024). Transgene-free CRISPR/Cas9-mediated gene editing through protoplast-to-plant regeneration enhances active compounds in *Salvia miltiorrhiza*. Plant Biotechnol. J..

[51] Zhang X., Xu G., Cheng C., Lei L., Sun J., Xu Y., Deng C., Dai Z., Yang Z., Chen X. (2021). Establishment of an *Agrobacterium*-mediated genetic transformation and CRISPR/Cas9-mediated targeted mutagenesis in Hemp (*Cannabis Sativa* L.). Plant Biotechnol. J..

[52] Zhou P., Chen H., Dang J., Shi Z., Shao Y., Liu C., Fan L., Wu Q. (2022). Single-cell transcriptome of *Nepeta tenuifolia* leaves reveal differentiation trajectories in glandular trichomes. Front. Plant Sci..

[53] Sheen J. (2001). Signal Transduction in Maize and Arabidopsis Mesophyll Protoplasts. Plant Physiol..

[54] Ma C., Peng S., Guo D., Zhu J., Wang Y. (2024). Isolation of plant protoplast and its application intransient transformation. J. Trop. Biol..

[55] Huang G. (2011). Advances in Protoplast Fusion Technology. J. Hunan Univ. Sci. Eng..

[56] Chen K., Chen J., Pi X., Huang L., Li N. (2023). Isolation, Purification, and Application of Protoplasts and Transient Expression Systems in Plants. Int. J. Mol. Sci..

[57] Li J., Liu L., Ding B., Yang H., Wu Q., Zhang Y. (2023). Research Progress on Isolation and Culture of Plant Protoplasts. Mol. Plant Breed..

[58] Li S., Zhao R., Ye T., Guan R., Xu L., Ma X., Zhang J., Xiao S., Yuan D. (2022). Isolation, purification and PEG-mediated transient expression of mesophyll protoplasts in *Camellia oleifera*. Plant Methods.

[59] Liu H., Sun P., Tong Y., Gao X., Tang Z., Fan G. (2024). Establishment of transient and stable gene transformation systems in medicinal woody plant *Acanthopanax senticosus*. Chem. Biol. Technol. Agric..

[60] Kang H.H., Naing A.H., Kim C.K. (2020). Protoplast Isolation and Shoot Regeneration from Protoplast-Derived Callus of *Petunia hybrida* Cv. Mirage Rose. Biology.

[61] Wang H., Wang W., Zhan J., Huang W., Xu H. (2015). An efficient PEG-mediated transient gene expression system in grape protoplasts and its application in subcellular localization studies of flavonoids biosynthesis enzymes. Sci. Hortic..

[62] Matchett-Oates L., Mohamaden E., Spangenberg G.C., Cogan N.O.I. (2021). Development of a robust transient expression screening system in protoplasts of *Cannabis*. In Vitro Cell. Dev. Biol.-Plant.

[63] Carqueijeiro I., Noronha H., Duarte P., Gerós H., Sottomayor M. (2013). Vacuolar Transport of the Medicinal Alkaloids from *Catharanthus roseus* is Mediated by a Proton-Driven Antiport. Plant Physiol..

[64] Beard K.M., Boling A.W.H., Bargmann B.O.R. (2021). Protoplast isolation, transient transformation, and flow-cytometric analysis of reporter-gene activation in *Cannabis sativa* L.. Ind. Crops Prod..

[65] Kang M., Vu A.H., Casper A.L., Kim R., Wurlitzer J., Heinicke S., Yeroslaviz A., Caputi L., O’Connor S.E. (2025). Single-cell metabolome and RNA-seq multiplexing on single plant cells. Proc. Natl. Acad. Sci. USA.

[66] Lin Z., Huang L., Yu P., Chen J., Du S., Qin G., Zhang L., Li N., Yuan D. (2023). Development of a protoplast isolation system for functional gene expression and characterization using petals of *Camellia oleifera*. Plant Physiol. Biochem..

[67] Azad M.A.K., Yokota S., Ishiguri F., Yoshizawa N. (2006). Plant regeneration from mesophyll protoplasts of a medicinal plant, *Phellodendron amurense* rupr. In Vitro Cell. Dev. Biol.-Plant.

[68] Geetha S.P., Nirmal Babu K., Rema J., Ravindran P.N., Peter K.V. (2000). Isolation of protoplasts from cardamom (*Elettaria cardamomum* Maton.) and ginger (*Zingiber officinale* Rosc.). J. Spices Arom. Crops.

[69] Li Z., Zhang B., Wu Q., Lv J., Zhao X., Pan Y., Guo W., Zhou H., Wang F., Luo H. (2023). Establishment of isolation and transient expression system forprotoplasts from chrysanthemum petals. Plant Physiol. J..

[70] Vessabutr S., Grant W.F. (1995). Isolation, culture and regeneration of protoplasts from birdsfoot trefoil (*Lotus corniculatus*). Plant Cell Tiss. Org..

[71] Li R., Du K., Zhang C., Shen X., Yun L., Wang S., Li Z., Sun Z., Wei J., Li Y. (2024). Single-cell transcriptome profiling reveals the spatiotemporal distribution of triterpenoid saponin biosynthesis and transposable element activity in *Gynostemma pentaphyllum* shoot apexes and leaves. Front. Plant Sci..

[72] Wei X., Zhang M. (2004). Protoplast fusion in *Dendrobium candidum* and *Gynostemma pentaphyllum*. Chin. Tradit. Herb. Drugs.

[73] Liu Y., Xue Y., Tang J., Chen J., Chen M. (2019). Efficient mesophyll protoplast isolation and development of a transient expression system for castor-oil plant (*Ricinus communis* L.). Biol. Futur..

[74] Li J., Jia J., Qi F. (1993). Plant regeneration from callus protoplasts of *Codonopsis pilosula*. J. Integr. Plant Biol..

[75] Yang X., Ma D., Jiang F., Chen N., Ding B., Jin L., Qian C., Ding Z. (2014). Protoplasts isolation, purification and plant regeneration of *Pinellia cordata*. Chin. J. Chin. Mater. Med..

[76] Zhou H., Zhou Z., Zhang J., Kan H., Yin M., Zhang H., Wang L., Zhao J., Ye J. (2026). Induction of Embryogenic Callus, Protoplast Isolation, and PEG-Mediated Transformation Protocols in *Eucommia ulmoides*. Plants.

[77] Nath J., Joshi S., Joshi R. (2025). Optimized protoplast isolation and PEG-mediated transient gene expression for high-altitude medicinal plant *Picrorhiza kurrooa*. Plant Physiol. Rep..

[78] Stelmach-Wityk K., Szymonik K., Jones A.M.P., Grzebelus E. (2025). A novel protocol for protoplast isolation, transfection, and culture in *Cannabis sativa* L.. BMC Plant Biol..

[79] Monthony A.S., Jones A.M.P. (2024). Enhancing Protoplast Isolation and Early Cell Division from Cannabis sativa Callus Cultures via Phenylpropanoid Inhibition. Plants.

[80] Guo Y., Bai J., Zhang Z. (2007). Plant regeneration from embryogenic suspension-derived protoplasts of ginger (*Zingiber officinale* Rosc.). Plant Cell Tiss. Org..

[81] Hou S.W., Jia J.F. (2004). Plant regeneration from protoplasts isolated from embryogenic calli of the forage legume *Astragalus melilotoides* Pall. Plant Cell Rep..

[82] Roberts S.C., Naill M., Gibson D.M., Shuler M.L. (2003). A simple method for enhancing paclitaxel release from *Taxus canadensis* cell suspension cultures utilizing cell wall digesting enzymes. Plant Cell Rep..

[83] Chand P.K., Davey M.R., Power J.B. (1990). Efficient plant regeneration from cell suspension protoplasts of the woody medicinal plant *Solanum dulcamara* L. (bittersweet, woody nightshade). Plant Cell Tiss. Org..

[84] Revilla M.A., Ochatt S.J., Doughty S., Power J.B. (1987). A generall strategy for the isolation of mesophtll protoplasts from deciduous fruit and nut tree species. Plant Sci..

[85] Wang J., Wang Y., Lü T., Yang X., Liu J., Dong Y., Wang Y. (2022). An Efficient and Universal Protoplast Isolation Protocol Suitable for Transient Gene Expression Analysis and Single-Cell RNA Sequencing. Int. J. Mol. Sci..

[86] Chen J., Wang J., Wu Q., Ma Y., Pei J. (2020). Protoplast and its application in molecular mechanism of qualityformation of traditional Chinese medicine. China J. Chin. Mater. Med..

[87] Ren R., Gao J., Lu C., Wei Y., Jin J., Wong S.M., Zhu G., Yang F. (2020). Highly Efficient Protoplast Isolation and Transient Expression System for Functional Characterization of Flowering Related Genes in *Cymbidium* Orchids. Int. J. Mol. Sci..

[88] Potrykus I., Shillito R.D. (1986). Protoplasts: Isolation, culture, plant regeneration. Methods Enzymol..

[89] Davey M.R., Anthony P., Power J.B., Lowe K.C. (2005). 2004 SIVB Congress Symposium Proceedings “Thinking Outside the Cell”: Plant Protoplast Technology: Status and Applications. In Vitro Cell. Dev. Biol.-Plant.

[90] Kim J.B., Bergervoet J.E.M., Raemakers C.J.J.M., Jacobsen E., Visser R.G.F. (2005). Isolation of protoplasts, and culture and regeneration into plants in *Alstroemeria*. In Vitro Cell. Dev. Biol.-Plant.

[91] Reed K.M., Bargmann B.O.R. (2021). Protoplast Regeneration and Its Use in New Plant Breeding Technologies. Front. Genome Ed..

[92] Yasugi S., Bajaj Y.P.S. (1989). Isolation and Culture of Orchid Protoplasts. Plant Protoplasts and Genetic Engineering I.

[93] Zhang C., Huang H., Cheng H., Wang G., Chen X., Zhong G., Li T., Deng W. (2024). Establishment andapplication of PEG-mediated protoplast genetic transformation system of *Cordyceps guangdongensis*. Guangdong Agric. Sci..

[94] Thomas A., Pujari I., Shetty V., Joshi M.B., Rai P.S., Satyamoorthy K., Babu V.S. (2016). *Dendrobium* protoplast co-culture promotes phytochemical assemblage in vitro. Protoplasma.

[95] Rao K.S., Prakash A.H. (1995). A simple method for the isolation of plant protoplasts. J. Biosci..

[96] Biswas S., Wahl N.J., Thomson M.J., Cason J.M., McCutchen B.F., Septiningsih E.M. (2022). Optimization of Protoplast Isolation and Transformation for a Pilot Study of Genome Editing in Peanut by Targeting the Allergen Gene *Ara h 2*. Int. J. Mol. Sci..

[97] Yao L., Liao X., Gan Z., Peng X., Wang P., Li S., Li T. (2016). Protoplast isolation and development of a transient expression system for sweet cherry (*Prunus avium* L.). Sci. Hortic..

[98] Stajič E. (2023). Improvements in Protoplast Isolation Protocol and Regeneration of Different Cabbage (*Brassica oleracea var. capitata* L.) Cultivars. Plants.

[99] Li Y., Wang X., Yang L., Yang B., Ning L., Dong Z. (2020). Effect of Enzymatic Hydrolysis Time on Yield and Viability of Protoplast in *Erigeron breviscapus*. J. Tianjin Agric. Sci..

[100] Tian Y., Liu M., Tang L., Zhang Y., Hang Y., Shangguan L., Zhang Y., Zhang M. (2023). Establishment of protoplasts transient expression system in *Pinellia ternata* (Thunb.) Breit. Biotechnol. Lett..

[101] Ahmed M.A.A., Miao M., Pratsinakis E.D., Zhang H., Wang W., Yuan Y., Lyu M., Iftikhar J., Yousef A.F., Madesis P. (2021). Protoplast Isolation, Fusion, Culture and Transformation in the Woody Plant *Jasminum* spp.. Agriculture.

[102] Barakat M.N., Cocking E.C. (1983). Plant regeneration from protoplast-derived tissues of *Linum usitatissimum* L. (Flax). Plant Cell Rep..

[103] Ahuja P.S., Hadiuzzaman S., Davey M.R., Cocking E.C. (1983). Prolific plant regeneration from protoplast-derived tissues of *Lotus corniculatus* L. (birdsfoot trefoil). Plant Cell Rep..

[104] Sakamoto S., Matsui K., Oshima Y., Mitsuda N. (2020). Efficient transient gene expression system using buckwheat hypocotyl protoplasts for large-scale experiments. Breed. Sci..

[105] Luo J., Mu Q., Gu Y. (1999). Protoplast culture and paclitaxel production by *Taxus yunnanensis*. Plant Cell Tiss. Org..

[106] Rao P.S., Ozias-Akins P. (1985). Plant regeneration through somatic embryogenesis in protoplast cultures of sandalwood (*Santalum album* L.). Protoplasma.

[107] Wu S., Morotti A.L.M., Yang J., Wang E., Tatsis E.C. (2024). Single-cell RNA sequencing facilitates the elucidation of the complete biosynthesis of the antidepressant hyperforin in St. John’s wort. Mol. Plant.

[108] Zhang S., Zhu C., Zhang X., Liu M., Xue X., Lai C., Xuhan X., Chen Y., Zhang Z., Lai Z. (2023). Single-cell RNA sequencing analysis of the embryogenic callus clarifies the spatiotemporal developmental trajectories of the early somatic embryo in *Dimocarpus longan*. Plant J..

[109] Wang G., Xia Z. (1983). Regeneration of Plantlets from *Solanum nigrum* L. Mesophyll Protoplasts. J. Integr. Plant Biol..

[110] Ren C., Tang X., Chen J., Wu Y., Wu Q., Wang L., Wang Q., Pei J. (2018). Cloning and Analysis of Promoter Regions of Flavonoid Biosynthesis Genes in *Safflower*. Plant Mol. Biol. Rep..

[111] Arya S., Liu J.R., Eriksson T. (1991). Plant regeneration from protoplasts of *Panax ginseng* (C.A. Meyer) through somatic embryogenesis. Plant Cell Rep..

[112] Xia G., Li Z., Guo G., Chen H. (1992). Direct somatic embryogenesis and plant regeneration from protoplasts of *Bupleurum scorzonerifolium* Willd. Plant Cell Rep..

[113] Wang J., Chen H. (1991). Plant regeneration from protoplast of *Peucedanum praeruptorum* Dunn. J. Integr. Plant Biol..

[114] Saxena P., Gill R., Rashid A., Maheshwari S.C. (1982). Plantlets from mesophyll protoplasts of *Solanum xanthocarpum*. Plant Cell Rep..

[115] Wang Q., Zhang M., Han M., Rong J., Peng W., Wang Y., Zhao Y., Lei X., Zhang J., Wang Y. (2025). Development of a transient expression system for *Panax ginseng* based on protoplast isolation from its embryoids. Hortic. Plant J..

[116] Shen J., Fu J., Ma J., Wang X., Gao C., Zhuang C., Wan J., Jiang L. (2014). Isolation, Culture, and Transient Transformation of Plant Protoplasts. Curr. Protoc. Cell Biol..

[117] Yang Z., Patra B., Li R., Pattanaik S., Yuan L. (2013). Promoter analysis reveals cis-regulatory motifs associated with the expression of the WRKY transcription factor *CrWRKY1* in *Catharanthus roseus*. Planta.

[118] Gou Y., Li Y., Bi P., Wang D., Ma Y., Hu Y., Zhou H., Wen Y., Feng J. (2020). Optimization of the protoplast transient expression system for gene functional studies in strawberry (*Fragaria vesca*). Plant Cell Tiss. Org..

[119] Meng R., Wang C., Wang L., Liu Y., Zhan Q., Zheng J., Li J. (2020). An efficient sorghum protoplast assay for transient gene expression and gene editing by CRISPR/Cas9. PeerJ.

[120] Pitzschke A., Persak H. (2012). Poinsettia protoplasts-a simple, robust and efficient system for transient gene expression studies. Plant Methods.

[121] Patra B., Pattanaik S., Schluttenhofer C., Yuan L. (2017). A network of jasmonate-responsive bHLH factors modulate monoterpenoid indole alkaloid biosynthesis in *Catharanthus roseus*. New Phytol..

[122] Zhao N., Li C., Yan Y., Wang H., Wang L., Jiang J., Chen S., Chen F. (2022). The transcriptional coactivator CmMBF1c is required for waterlogging tolerance in *Chrysanthemum morifolium*. Hortic. Res..

[123] Yuan J., Li D., Liang Y., Meng Y., Li L., Yang L., Pei M., Feng L., Li J. (2024). An optimum study on the laser scanning confocal microscopy techniques for BiFC assay using plant protoplast. Bot. Stud..

[124] Xi Z., Li Y., Liu S., Wang D., Guo J., Xian B., Rao K., Chen C., Peng Y., Zhou Y. (2025). Functional analysis and molecular characterization of UGT95A2, a specialized glycosyltransferase for flavonoid 3′-O-glycosylation in *Carthamus tinctorius* L.. Plant J..

[125] Syombua E.D., Zhang Z., Tripathi J.N., Ntui V.O., Kang M., George O.O., Edward N.K., Wang K., Yang B., Tripathi L. (2020). A CRISPR/Cas9-based genome-editing system for yam (*Dioscorea* spp.). Plant Biotechnol. J..

[126] Liu K., Sun B., You H., Tu J., Yu X., Zhao P., Xu J. (2020). Dual sgRNA-directed gene deletion in basidiomycete *Ganoderma lucidum* using the CRISPR/Cas9 system. Microb. Biotechnol..

[127] Lv Z., Chen W., Fang S., Dong B., Wang X., Zhang L., Xue J., Chen W. (2024). Targeted mutagenesis in Arabidopsis and medicinal plants using transposon-associated TnpB. J. Integr. Plant Biol..

[128] Cai R., Chai N., Zhang J., Tan J., Liu Y., Zhu Q., Zeng D. (2025). CRISPR/Cas system-mediated transgene-free or DNA-free genome editing in plants. Theor. Appl. Genet..

[129] Huo G., Zhang X., Tian S., Li J. (2025). Current Progress and Applications of CRISPR/Cas12a Gene Editing Technology in Plants. Biotechnol. Bull..

[130] Zhang R., Zheng Z., Li G., Zheng X., Su L., Yuan X., Li T., Tan J., Zeng D., Zhang S. (2026). Plant base editing: A decade of progress and future applications. aBIOTECH.

[131] Yu H., Feng X., Zheng X., Wang X., Zheng W., Zhang Z., Jiang Y., Yang R., Zhang L., Zhong Z. (2026). Enhancing Metabolic Engineering in Medicinal Plants Through Prime Editing. Plant Biotechnol. J..

[132] Hsieh J.W.A., Wu F.H., Yang D.X., Wu A.E., Liu C.A., Chen C.H., Lin S.Z., Lin Y.C.J., Lin C.S. (2026). Protoplast-Based Functional Genomics and Genome Editing: Progress, Challenges and Applications. Plant Cell Environ..

[133] Golla D.A., Sun C., Haugh L., Straub N., Gao X. (2026). Advances in multiplex precision genome editing in eukaryotic and prokaryotic systems. Curr. Opin. Biotechnol..

[134] Mo Y., Jiao Y. (2022). Advances and applications of single-cell omics technologies in plant research. Plant J..

[135] Lv Z., Jiang S., Kong S., Zhang X., Yue J., Zhao W., Li L., Lin S. (2024). Advances in Single-Cell Transcriptome Sequencing and Spatial Transcriptome Sequencing in Plants. Plants.

[136] He Q., E Y., Li R. (2020). Single-Cell Sequencing Technology and Its Research Progress in Plant cells. Chin. J. Cell Biol..

[137] Yu C., Hou K., Zhang H., Liang X., Chen C., Wang Z., Wu Q., Chen G., He J., Bai E. (2023). Integrated mass spectrometry imaging and single-cell transcriptome atlas strategies provide novel insights into taxoid biosynthesis and transport in *Taxus mairei* stems. Plant J..

[138] Bawa G., Liu Z., Yu X., Qin A., Sun X. (2022). Single-Cell RNA Sequencing for Plant Research: Insights and Possible Benefits. Int. J. Mol. Sci..

[139] Shaw R., Tian X., Xu J. (2021). Single-Cell Transcriptome Analysis in Plants: Advances and Challenges. Mol. Plant.

[140] Wang K., Zhao C., Xiang S., Duan K., Chen X., Guo X., Sahu S.K. (2023). An optimized FACS-free single-nucleus RNA sequencing (snRNA-seq) method for plant science research. Plant Sci..

[141] Longo S.K., Guo M.G., Ji A.L., Khavari P.A. (2021). Integrating single-cell and spatial transcriptomics to elucidate intercellular tissue dynamics. Nat. Rev. Genet..

[142] Liu S., Li X., Zhu J., Jin Y., Xia C., Zheng B., Silvestri C., Cui F. (2024). Modern Technologies Provide New Opportunities for Somatic Hybridization in the Breeding of Woody Plants. Plants.

[143] Huo L., Xiang F., Xia G. (2000). Asymmetric somatic hybridization between *Peucedanum terebinthaceum* Fisch and *Bupleurum scorzonerifolium* wild. J. Shandong Univ. (Nat. Sci).

[144] Guan Q., Guo Y., Wei Y., Meng F., Zhang Z. (2010). Regeneration of somatic hybrids of ginger via chemical protoplast fusion. Plant Cell Tiss. Org..

[145] Zhang F., Wang P., Ji D., Kang G., Xiang F. (2011). Asymmetric somatic hybridization between *Bupleurum scorzonerifolium* Willd. and *Taxus chinensis* var. *mairei*. Plant Cell Rep..

[146] Wang J., Zhou P., Li C., Liang Y., Liu G., Yang S., Xiao Y., Zhao Y. (2024). Progress on medicinal plant regeneration and the road ahead. Med. Plant Biol..

[147] Castelblanque L., García-Sogo B., Pineda B., Moreno V. (2009). Efficient plant regeneration from protoplasts of *Kalanchoe blossfeldiana* via organogenesis. Plant Cell Tiss. Org..

[148] Xu X.H., Davey M.R. (1983). Shoot regeneration from mesophyll protoplasts and leaf explants of *Rehmannia glutinosa*. Plant Cell Rep..

[149] Maqsood M., Mujib A., Tonk D., Abdin Z.M. (2012). Protoplast Isolation, Culture and Plant Regeneration in *Catharanthus roseus* (L.) G. Don via Somatic Embryogenesis. Curr. Biotechnol..

[150] Ratushnyak Y.I., Rudas V.A., Piven N.M. (1990). Regeneration of *Lycium barbarum* L. plants from leaf tissue, callus culture and callus protoplasts. Plant Cell Rep..

[151] Cao W., Cai X. (2012). Review of Plant Protoplast Recalcitrance and Its Physiological and Genetic Bases. Hubei Agric. Sci..

[152] He Y., Xu L., Liu Q. (2025). The cellular epigenetic blueprint of plant regeneration. Curr. Opin. Plant Biol..

[153] Papadakis A.K., Roubelakis-Angelakis K.A. (2002). Oxidative stress could be responsible for the recalcitrance of plant protoplasts. Plant Physiol. Biochem..

[154] Bairu M.W., Aremu A.O., Van Staden J. (2010). Somaclonal variation in plants: Causes and detection methods. Plant Growth Regul..

